# Adapting the ADVANCE group program for digitally-supported delivery to reduce intimate partner violence by men in substance use treatment: a feasibility study

**DOI:** 10.3389/fpsyt.2023.1253126

**Published:** 2024-01-24

**Authors:** Gail Gilchrist, Sandi Dheensa, Amy Johnson, Juliet Henderson, Polly Radcliffe, Georges Dwyer, Richard Turner, Kate Thomson, Cat Papastavrou Brooks, Beverly Love, Zohra Zenasni, Cassandra Berbary, Ben Carter, Steve Parrott, Jinshuo Li, Caroline Easton, Ciara Bergman, Gene Feder, Elizabeth Gilchrist

**Affiliations:** ^1^National Addiction Center, Institute of Psychiatry, Psychology and Neuroscience, King’s College London, London, United Kingdom; ^2^Population Health Sciences, Bristol Medical School, University of Bristol, Bristol, United Kingdom; ^3^School of Health in Social Science, Department of Clinical Psychology, Center for Psychological Therapies, University of Edinburgh, Edinburgh, United Kingdom; ^4^Department of Biostatistics and Health Informatics, Institute of Psychiatry, Psychology and Neuroscience, King’s College London, London, United Kingdom; ^5^College of Health Sciences and Technology, Rochester Institute of Technology, Rochester, NY, United States; ^6^Department of Health Sciences, University of York, York, United Kingdom; ^7^Respect, London, United Kingdom

**Keywords:** intimate partner violence, substance use treatment, perpetrator, remote delivery, integrated intervention, blended interventions, digital interventions, ADVANCE-D

## Abstract

**Introduction:**

COVID-19 restrictions created barriers to “business as usual” in healthcare but also opened the door to innovation driven by necessity. This manuscript (1) describes how ADVANCE, an in-person group perpetrator program to reduce intimate partner violence (IPV) against female (ex)partners by men in substance use treatment, was adapted for digitally-supported delivery (ADVANCE-D), and (2) explores the feasibility and acceptability of delivering ADVANCE-D to men receiving substance use treatment.

**Methods:**

Firstly, the person-based approach and mHealth development framework were used to iteratively adapt ADVANCE for digitally-supported delivery including conceptualization, formative research, and pre-testing. Then, a non-randomized feasibility study was conducted to assess male participants’ eligibility, recruitment, and attendance rates and uptake of support offered to their (ex)partners. Exploratory analyses on reductions in IPV perpetration (assessed using the Abusive Behavior Inventory; ABI) and victimization (using the revised ABI; ABI-R) at the end of the program were performed. Longitudinal qualitative interviews with participants, their (ex)partners, and staff provided an understanding of the program’s implementation, acceptability, and outcomes.

**Results:**

The adapted ADVANCE-D program includes one goal-setting session, seven online groups, 12 self-directed website sessions, and 12 coaching calls. ADVANCE-D includes enhanced risk management and support for (ex)partners. Forty-five participants who had perpetrated IPV in the past 12 months were recruited, forty of whom were offered ADVANCE-D, attending 11.4 (SD 9.1) sessions on average. Twenty-one (ex)partners were recruited, 13 of whom accepted specialist support. Reductions in some IPV perpetration and victimization outcome measures were reported by the 25 participants and 11 (ex)partners interviewed pre and post-program, respectively. Twenty-two participants, 11 (ex)partners, 12 facilitators, and 7 integrated support service workers were interviewed at least once about their experiences of participation. Overall, the program content was well-received. Some participants and facilitators believed digital sessions offered increased accessibility.

**Conclusion:**

The digitally-supported delivery of ADVANCE-D was feasible and acceptable. Remote delivery has applicability post-pandemic, providing greater flexibility and access. Given the small sample size and study design, we do not know if reductions in IPV were due to ADVANCE-D, time, participant factors, or chance. More research is needed before conclusions can be made about the efficacy of ADVANCE-D.

## 1 Introduction

Intimate partner violence (IPV) perpetration involves any behavior causing physical, sexual, or psychological harm, including aggression, sexual coercion, psychological, and financial abuse and coercive control (1). While IPV occurs in all relationships (1–4), women experience greater injury (1, 5, 6) and are six times more likely to be murdered by their intimate partner than men (7). Multiple risk factors contribute and interact at individual, relationship, community, and societal levels to increase the likelihood of IPV perpetration, including substance use, mental health problems, adverse childhood experiences, anger, hostility, poor executive function, low empathy, relationship conflicts, and holding sexist attitudes (8–12). Many risk factors are elevated among men who have substance use problems (13, 14), potentially explaining the higher prevalence of IPV perpetration among this group compared to the general population (8, 14–16) and highlighting that targeted perpetrator programs are needed (14, 17, 18). Limited evidence shows what works to reduce IPV perpetration by men who use substances (19), but programs that include trauma and substance use components show promising results and/or greater reductions in perpetration (20, 21). Regardless, men in substance use treatment are underserved by perpetrator programs (22, 23) and often considered unsuitable for such programs due to their substance use (24). Men who use substances are also most likely to drop out of perpetrator programs (14, 23, 24), suggesting alternative and tailored approaches are required. We developed the ADVANCE perpetrator group program (25–27) to address the complex interplay between substance use and IPV perpetration in the context of intoxication, withdrawal, craving and acquiring substances (28–30), neglected in other programs. Our research highlighted the importance of addressing sexual jealousy and entitlement, the wider dynamics of power and control, and psychological vulnerabilities (29–31).

Pre-pandemic there was little evidence about remotely delivering perpetrator programs online (32). One study highlighted enhanced accessibility and flexibility, but that access to hardware, broadband, and private space posed challenges (32). Early in the pandemic, the limited guidance on remote delivery focused on reducing short-term risk via safety-planning, including for co-habiting survivors and children, and de-escalation (33, 34). As remote delivery of substance use and perpetrator programs continued, the need to carefully and specifically adapt perpetrator programs and integrated survivor support (35) for digital delivery, drawing on evidence and theory became clear, particularly because IPV and substance use reportedly increased during lockdown (36–40). We therefore adapted our in-person ADVANCE group perpetrator program (25) for remote delivery.

## 2 Aims

We present (1) the process of iteratively adapting our in-person group for digitally-supported delivery (ADVANCE-D) for men in United Kingdom substance use treatment settings, (2) qualitative and quantitative findings from a multi-center, non-randomized feasibility and acceptability study, with a nested process evaluation, and (3) preliminary outcomes on IPV perpetration and victimization.

## 3 Methodology

### 3.1 Iterative adaptation of ADVANCE

#### 3.1.1 Overview of methodology

The person-based approach (41, 42), and mHealth development framework (43) were used to iteratively adapt ADVANCE for digitally-supported delivery during August 2020–June 2021, involving:

- Conceptualization: Scoping reviews of literature and emerging guidance on delivering psychosocial interventions remotely and/or digitally were undertaken. Given the timeframe of the adaptation, only evidence published pre pandemic or published early into the pandemic (in 2020) was included in the literature reviews. Through our existing Learning Alliances, professionals from criminal justice, substance use and IPV perpetrator and survivor organizations were consulted to identify remote delivery and risk management practices.
- Formative research: Multiple rounds of consultations with key stakeholders—staff from substance use services, Learning Alliance members, men who used substances, and IPV victims/survivors—were conducted and proposed approaches and materials were grounded in their reality and practice. Following the conceptualization phase, it was decided that a blended approach would be used to deliver ADVANCE based on the available evidence, including online individual and group sessions, website sessions, and coaching calls. The website sessions would use an “online coach” to “deliver” ADVANCE material from the original intervention, and guide men through sessions. It was also decided that an (ex)partner’s version of the website, for (ex)partners to view would be created. Nominal group technique (44) was used to reach consensus with stakeholders from the two Learning Alliances on the delivery model options and on the look and feel of the online coach and of the website sessions. Six men with lived experience of using IPV and/or substance use (from here on referred to as men with lived experience) were consulted on the paper prototype of the website sessions, their experiences of using technology and ideas around how to enhance engagement (45). These consultations helped us understand how we could best deliver and communicate digital content. Comments were fed back to the developers and designers at Rochester Institute of Technology to inform the development of a digital prototype of the first three website sessions including the online coach.
- Pre-testing: Individual consultations (using videocalls) were held with eight male substance use treatment service users and four female IPV survivors on the male and (ex)partner website prototypes, respectively. They were given a link to a test website, asked by a researcher to share their screen and to “think aloud” while using the website (41). Their views about intervention flow, structure and design were recorded. Decisions on whether to modify were based on whether changes were likely to impact acceptability, feasibility, persuasiveness, motivation, and engagement (41) using MoSCoW (must-have, should-have, could-have, and will not-have) prioritization criteria (46). Pre-testing was completed remotely using a secure online meeting platform due to COVID-19 restrictions.

Below, we combine the findings from these three stages of adaptation to explain key decisions made about: mode of delivery, website design and features, addressing digital poverty (i.e., being unable to interact with the online world due to financial or geographical barriers, or not having the skills to do so), therapeutic alliance and group size, and enhanced risk and safety management. We then give an overall description of the adapted program (ADVANCE-D). The consultation and user testing were conducted in the earlier stages of the COVID-19 restrictions when staff and service users may have been less familiar with using online programs.

#### 3.1.2 Key decisions made about the adapted version

##### 3.1.2.1 Choosing a blended mode of delivery

**Figure 1:**
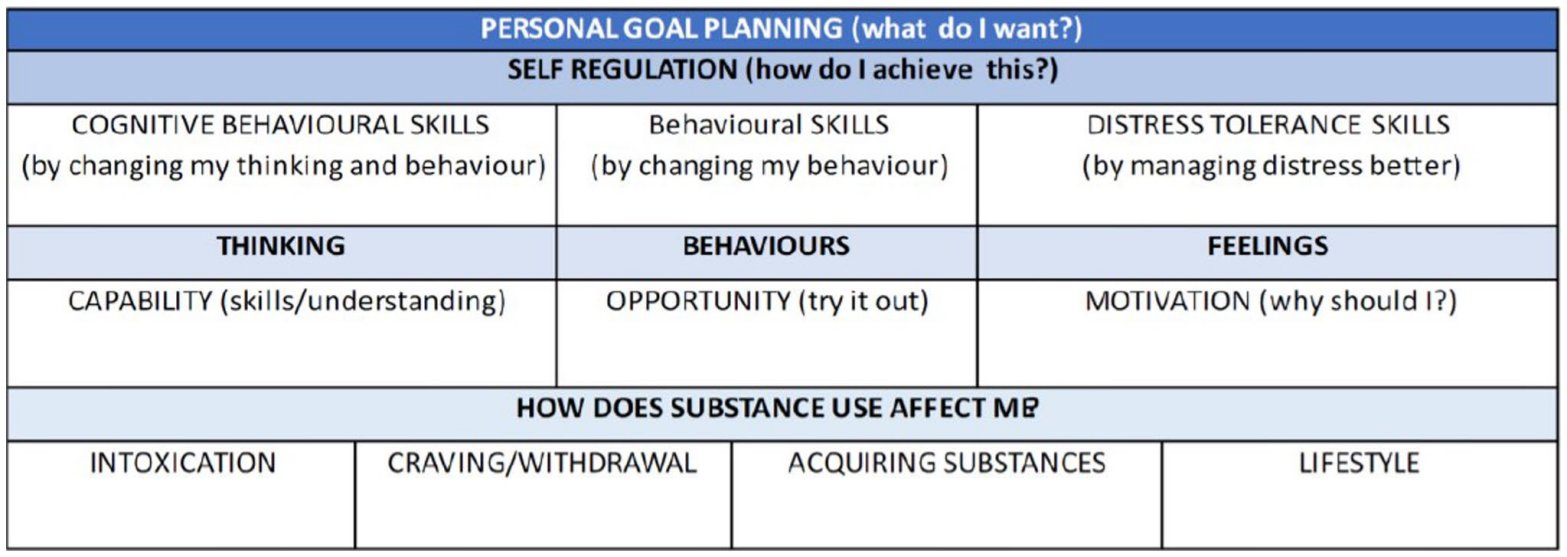
Theory guiding the ADVANCE intervention.

**Figure 2:**
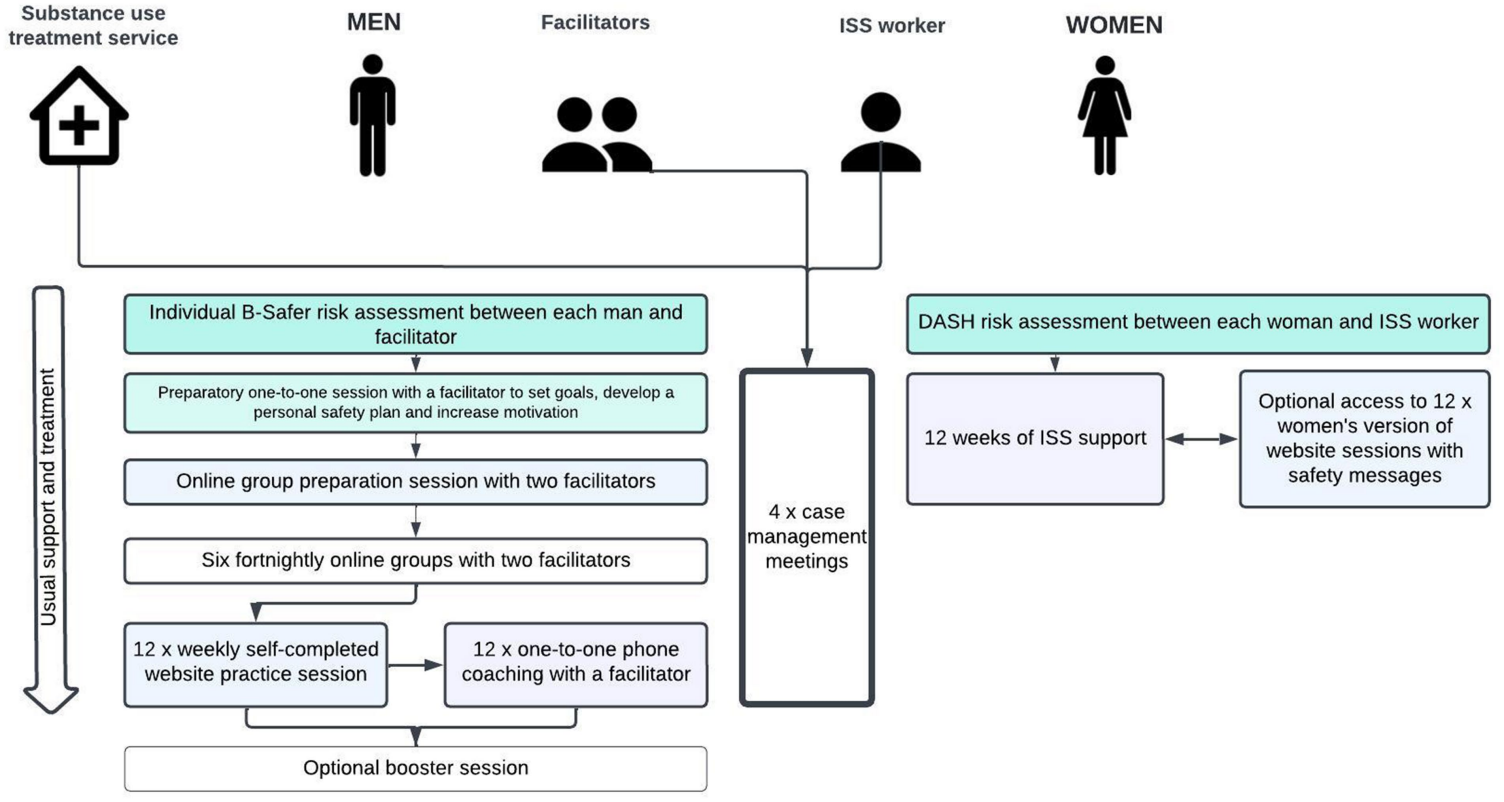
Overview of ADVANCE-D intervention.

Our early consultations with key stakeholders at the outset of the pandemic highlighted that many criminal justice and substance use services decided not to migrate to online delivery, opting for telephone check-in sessions instead, to monitor risk, safety, relationship status, and substance use, and to signpost people to contact other appropriate services, or make necessary referrals. Trials of guided (patient communicates with a health care professional) and unguided internet-based cognitive behavioral therapy (iCBT) pre-COVID-19 found that these approaches were acceptable to patients and effective in reducing depressive symptoms (47) and alcohol use (48). Guided iCBT was found to be more effective than unguided iCBT (47, 48). Blended care combining iCBT and face-to-face sessions with a therapist also shows positive outcomes (49), comparable to face-to-face CBT alone (50). Other studies have shown significant improvements in outcomes for programs that combine self-directed support with telephone facilitation (51, 52). We chose a blended digitally-supported delivery as the best approach to deliver ADVANCE-D remotely, combining online groups, self-complete website sessions to practice what was learned in the groups and phone or videocall coaching/support from a facilitator (52–54). Figure 1 summarizes the ADVANCE-D program theory and Figure 2 presents the adapted model. The offer of personal contact and support to promote use of the material and monitor risk was based on evidence that this approach showed better outcomes than online interventions without such support (47–54). This was also requested by men with lived experience and key stakeholders.

In one study, men attending behavior change programs suggested that a digital intervention could encourage men to consider the impact of their behavior on their families, particularly their children (55). The ADVANCE-D website sessions were designed to repeat and build on the content of the group sessions, giving participants this chance. The men we consulted wanted the website sessions to include recaps, avoid quizzes or tests, take <30 min to complete and provide “recognition” for completing sessions. These were all included in the website sessions. They also requested an introduction session to learn how to use the technology and to have reminders of when to complete the website sessions which were also incorporated into delivery.

##### 3.1.2.2 Website design and features

Static educational materials (i.e., unguided self-help content), lead to low engagement (53). Factors that increase engagement include a sense of “connectedness”—that is, interactivity, personalization through tailored content and/or feedback, such as by facilitators exploring ways that participants can incorporate learning into their day-to-day, feeling informed, supported, and understood, and getting reminder emails to complete sessions (56–58). ADVANCE-D website sessions incorporated personalization—the online coach “led” interactive sessions (e.g., participants were asked to input how they were feeling and the online coach acknowledged if they were feeling low). Sessions integrated film clips and multiple-choice question and answers, to increase engagement. “Try it out” activities (i.e., “homework” was set at the end of each website session) encouraged participants to incorporate learning into their daily lives. The sessions were followed up with phone or videocall coaching and tailored support from a facilitator to promote engagement and monitor risk.

Similar to another patient feedback study of avatar-based interactive technologies for co-occurring substance use and IPV, our user consultation wanted the digital coach to look like a real-life professional rather than a cartoon (59).

Disclosure of sensitive information (including drug use and IPV victimization) was more likely to avatars than face-to-face (60). Men with lived experience requested that all content spoken by the online coach was also provided in text. Evidence also suggests that new visual information should be explained with audio rather than text (61).

Think-aloud videocall interviews to review the digital prototypes with men with lived experience suggested changes to the website for ease-of-use, clarity and visual impact. They requested the inclusion of a progress bar to allow the user to know how much of each session had been completed. Ability to monitor progress also influences engagement (62, 63). These were all implemented as well as participants having the ability to take a break from the website session and pick up where they left off when they logged in again.

##### 3.1.2.3 Addressing digital poverty

Initial consultation with service users and service providers highlighted that access to both hardware and mobile data could hinder engagement with ADVANCE-D. This was supported by research in United Kingdom community drug treatment services that found only 57% of the 83% of clients who owned a mobile phone had a smartphone, and that 72% of clients used pay-as-you-go contracts (64). Therefore, providing tablets and data to participants in this study was required to facilitate engagement. Service users and providers also highlighted a potential lack of digital literacy and suggested providing knowledge about how to use the software and access to technical support. To address digital poverty and enhance digital literacy (64–66), we sent ready to use tablets (preference from our consultation over smartphones) with front-facing cameras with 4G connectivity and headphones to participants, and research staff provided technology support by phone or email, alongside a “how to guide” to use the technology and platform. Men with lived experience suggested that contingency management should be linked to pro-social activities (e.g., cinema, gym membership etc.), but such activities were not always possible during COVID-19 restrictions. They also highlighted that the cost of mobile data may contribute to lower engagement. Therefore, we later decided to provide free monthly mobile data contingent on attendance, and men who completed ADVANCE-D were permitted to keep the tablet after the study ended as a further incentive. This decision was supported during a later consultation with people with lived experience and service providers.

##### 3.1.2.4 Therapeutic alliance and group size

Facilitators of online groups must pay attention to building therapeutic alliance and to group process, as these are linked to positive treatment outcomes (67). Participant cohesion can be developed through group agreements. Paying attention to social cues and signs of emotionality (facial expression, tone of voice, or body language) and asking more questions than usual to clarify responses and reactions can help build therapeutic alliance online (53). These techniques, as well as attending to group dynamics, giving equal time and attention to all members of the group and promoting positive, respectful communication, were built into the ADVANCE-D model, and were emphasized in training and integrity support meetings. Studies of therapeutic groups, comprising 5–13 participants, suggest group size does not predict outcomes (68). Slightly smaller groups are more appropriate for participants with learning disabilities or behavioral problems or for CBT-based groups due to level of skills imparted (69). Therefore, ADVANCE-D sought to recruit between 6 and 12 participants for each group.

##### 3.1.2.5 Enhanced risk and safety management

Concerns around delivering perpetrator programs online included lack of face-to-face cues, difficulty in managing complex group dynamics and limitations in modeling of gender equality via co-facilitation (70). Our consultations with key stakeholders and guidance available at the time (33, 34, 70), emphasized the importance of a group agreement, including rules on not misusing technology (e.g., for illegal or abusive purposes), keeping cameras on to allow facilitators to gauge potential intoxication and substance use, personal identifying items (e.g., photos of children) to be out of range, and not attending/completing sessions intoxicated. Participants were asked to complete and join sessions in a private room where they could not be overheard and to use the headphones provided if necessary. Facilitators were asked to pay attention to participants’ emotional wellbeing, substance use, home living situation, and to act on any change in risk related factors.

ADVANCE-D was structured to ensure that group members were not left feeling triggered to enact abuse or emotionally dysregulated after online groups or website sessions. Check outs, one-to-one coaching calls and specific ADVANCE-D materials, including use of self-soothing sensory items and relaxation techniques (used in the original ADVANCE program) were identified as strategies to help manage difficult thoughts or emotions. We decided that scheduling times for coaching calls with facilitators after completing website sessions would encourage participants to refrain from substance use before/during a session and help manage risk.

Integrated support service (ISS) workers completed the Domestic Abuse, Stalking, Harassment and Honor Based Violence Assessment (DASH) (71) with participants’ (ex) partners. Facilitators completed the ADVANCE-D risk assessment form based on the Brief Spousal Assault Form for the Evaluation of Risk (B-SAFER) (72) with participants to assess suitability and manage and mitigate risk. Both had regular supervision and four case management meetings were held between them. The clinical risk lead provided program management fortnightly support meetings online to all professionals to ensure fidelity to the program, where ADVANCE-D content, group process, online delivery, risk and safety management were all covered. This helped facilitators feel confident and supported in their roles (35).

In order not to privilege group participants over their (ex)partners, we decided to develop a (ex)partner version of the participants’ website, containing safety messages for (ex)partners. (Ex)partners could view but not interact with the ADVANCE-D website content. They were offered password-protected smartphones with 4-month mobile data (the duration of the research study), addressing the risk of their own phone activity being monitored or intercepted by the perpetrator and allowing them to view the safety messages. During the pre-testing of ADVANCE-D, female survivors consulted about website appropriateness found the safety messages for women acceptable and welcomed the opportunity to access the website, noting that other perpetrator programs do not share content with survivors. An exit button was provided that enabled (ex)partners to exit the site immediately.

ADVANCE-D was therefore iteratively developed taking all of the above-mentioned evidence, consultation and best practice into consideration.

#### 3.1.3 Overall description of ADVANCE-D, the adapted program

In the adapted program, ADVANCE-D, the content and underpinning theory remained the same as the original ADVANCE group program which targets individual risks for IPV, including substance use, poor emotional regulation, and poor stress-coping, and teaches participants how to reduce these risks by promoting self-regulation (ability to alter a response or override a thought, feeling, or impulse) (73) and personal goal setting (Figure 1). Contingency management (74) was used to enhance engagement and attendance, building on the “Good Lives Model” (75) underpinning the intervention. Although delivered in a different format, it remained fundamentally the same in that it relied on positive therapeutic alliance, well facilitated group process and strengths-based change. ADVANCE-D comprised 32 sessions, delivered remotely by two trained facilitators over 14 weeks. It included an individual session with a facilitator to set goals, develop a personal safety plan and increase motivation; a preparatory online group to prepare participants for taking part in ADVANCE-D; six fortnightly online groups, 12 self-completed website sessions and 12 coaching calls to account for best practice in terms of monitoring and managing risk and safety and increasing skills and knowledge (Figure 2). In the 2 weeks following each group, participants would complete two practice website sessions each followed by a coaching call. Participants advised the facilitator when they were intending to complete the session so that the coaching call could be booked in to follow. The 12 weekly self-directed website sessions were guided by an online coach to recap and practice skills learned in the online group sessions. The online coach verbalized the content and the text also appeared on screen, so participants had the option to listen to the coach and/or read the text. Coaching calls were delivered by a facilitator to go over the materials in the previous group and website session, check in with the participant around their relationship and substance use, especially any change in risk, and continue to enhance motivation and revisit goals. A refresher session was offered 1 month after the last online group took place.

(Ex)partners are offered support around IPV from an ISS worker and access to the website sessions (see section 3.2). The (ex)partner’s version of the website has additional information to support them to stay safe.

### 3.2 Multi-center non-randomized feasibility study with nested process evaluation

#### 3.2.1 Overview of methodology

A feasibility study was conducted in substance use treatment to assess eligibility, recruitment, follow-up, adverse events and uptake rates of ADVANCE-D for participants and ISS for their (ex)partners. We also determined acceptability for participants by assessing attendance and completion rates for participants, therapeutic alliance and ratings for each website session, and multiple perspectives data (male and female participants, staff) collected via brief semi-structured interviews at up to four points in a qualitative longitudinal process evaluation (76). As program outcomes to determine preliminary efficacy, we assessed IPV perpetration by participants and experience of IPV victimization by (ex)partners at baseline and the end of the program. This mixed methods approach can help understand the intervention’s implementation and outcomes over time. Ethics approval was granted by Yorkshire and The Humber-Sheffield Research Ethics Committee on January 25, 2021 (Reference: 19/YH/0445).

#### 3.2.2 Participants, eligibility, and recruitment for feasibility study

We aimed to recruit 60 participants already engaged in community substance use treatment in London, the West Midlands, the South West, South Wales and Lothian (United Kingdom) (77). ADVANCE-D was offered to participants who “volunteered” for the program rather than being court-mandated to attend. Participants were screened during 06/21–11/21 for eligibility against the inclusion criteria by substance use treatment staff or researchers, first remotely and once restrictions eased, in person at substance use treatment services or online groups. Recruitment flyers were also emailed to potential participants by staff inviting them to contact researchers for information. Participants were eligible if they had perpetrated any IPV in the past 12 months assessed using the Abusive Behavior Inventory (ABI) (78) toward a female (ex)partner (i.e., scored positive to one of the 29 items) with whom they still had contact in the past 4 months and agreed to provide her contact details so that she could be invited to take part in the research by a researcher, offered support by an ISS worker and could be contacted and safeguarded if any risk relevant issues arose (79, 80). A trained facilitator then assessed the suitability of those eligible for ADVANCE-D using an adapted version of the B-SAFER (72). Men who were assessed as low to medium risk of re-abusing partners without intervention were suitable to attend the program, while those assessed as high risk were not. Those attending a perpetrator program, who had an order preventing them from contacting their (ex)partner or where there were other safety concerns were not eligible.

After participants were recruited and had provided the contact details of their (ex)partners, researchers texted or emailed their (ex)partners with brief information about the study and then contacted them to invite them to participate in the research. They were also advised that regardless of whether they agreed to take part, they would be contacted and offered support by an ISS worker. The ISS worker also completed the DASH (71) risk assessment with (ex)partners. Support focused on the needs of the individual but could include developing a safety plan, signposting after a needs assessment and counseling. The ISS worker could also offer to support (ex)partners in viewing the website and safety messages. Potential participants were given an information sheet and the opportunity to ask any questions. Informed eConsent or written consent was required prior to conducting a baseline interview.

#### 3.2.3 Data collection and outcomes

Seven researchers (five women, two men) administered quantitative questionnaires (see section 4.1.4) and conducted qualitative interviews. Only female researchers collected data from men’s current or ex-partners, while both male and female researchers interviewed participants. Different researchers interviewed the male participant and (ex)partner from each dyad to ensure that no information was inadvertently shared (81). The qualitative interviews with facilitators and ISS workers were conducted by a researcher not responsible for managing that research site to avoid bias. All interviews were conducted by telephone or videocall.

Changes in outcomes for participants and their (ex)partners were evaluated pre- and post the ADVANCE-D program. Only details of IPV and therapeutic alliance measures are presented in this manuscript. Incidents of IPV were not reported to the police by researchers. If severe IPV was reported [i.e., behaviors associated with a higher likelihood of a lethal outcome as defined by the Danger Assessment (82) including burning, punching, strangulation, or sexual violence], researchers enacted their duty of care by reporting such incidents to substance use treatment service or the ISS staff who made further decisions to report these incidents to police or social services to deliver supportive services based on services’ safeguarding protocols and legal requirements. Participants were advised of these limitations to confidentiality during the consent process, including a verbal and written explanation of this in the participant information sheet. Details of the full study methods are described elsewhere (76).

Intimate partner violence was assessed during the past 4 months using various measures. For participants, the 29-item ABI was administered to measure the frequency of the perpetration of physical (12 items, two of which assess sexual abuse) and psychological abuse (17 items) (78). For (ex)partners, the 25-item Abusive Behavior Inventory Revised (ABI-R) measured experiences of physical (13 items), psychological (nine items), and sexual abuse (three items) victimization (83). Items were scored from 1 (never) to 5 (very frequently), with higher subscale scores and total score indicative of greater abuse. Four adapted questions from the 24-item Revised Controlling Behaviors Scale (CBS-R) measured the use and experience of controlling behaviors (84) (e.g., want to know where your partner went and who they spoke to when not together). Response options ranged from 0 (never) to 4 (always). Two questions from a non-validated scale on the use of technology-facilitated abuse were included (85) (e.g., “Used mobile technology to check her location”). Total scores ranged from 2 (never) to 10 (very frequently). Four questions from a non-validated scale assessed the use of children against a partner (86) (e.g., “Asked the children to report on what she is doing or where she has been”), with total scores ranging from 4 (never) to 20 (very frequently). One item enquired about the frequency of stopping/being stopped by a partner from leaving the house against their will, scored from 1 (never) to 5 (very frequently). Two questions about stalking behaviors, scoring from 1 (never) to 5 (very frequently) were included. In all cases, the higher the total score, the greater the frequency of perpetrating or experiencing the behavior.

The 12-item Working Alliance Inventory applied to Virtual and Augmented Reality (WAI-VAR) (87) and the 12-item patient version of the California Psychotherapy Alliance Scale-Short Form (CALPAS-P Short Form) (88) assessed therapeutic alliance for participants at follow-up only. With the original author’s permission, we changed “virtual environment” to website in the WAI-VAR. Items on the WAI-VAR are scored from 1 (never) to 7 (always) for each subscale: Goals, Tasks, and Bonds. Total score ranges from 12 to 84, with higher scores suggesting a stronger working alliance. The CALPAS-P Short Form uses four subscales: the patient working capacity, patient commitment, working strategy consensus, and therapist understanding and involvement. Participants are asked to describe the extent to which each item describes their experience from 1 (not at all) to 7 (very much so). The total score is the mean of these four subscales.

Up to four longitudinal qualitative interviews were conducted during ADVANCE-D as part of the process evaluation to capture information about acceptability: views about the intervention, changes and impact over time for participants and their (ex)-partners, and staff’s experience of delivering ADVANCE-D. The interviews corresponded with completion of 4 weeks (to explore motivation, experience of welcome to group, online group 1 and website sessions 1 and 2), 8 weeks (experience of online groups 2 and 3, and website sessions 3–6, any behavior change), 12 weeks (experience of online groups 4 and 5, and website sessions 7–10, any behavior change), and 14 weeks (experience of online group 6, and website sessions 11–12, any behavior change) of the program. Therefore, interviews were only completed with men who remained engaged in the program at these pre-defined times. Participants and (ex)partner participants were reimbursed (£10 voucher) for each questionnaire and qualitative interview (up to a total of £60 vouchers, if baseline and follow-up questionnaire and 4 qualitative interviews were completed). Participants completed a rating scale online after completion of each website session.

Summary statistics were estimated to quantify relevant feasibility and acceptability parameters. In addition, scores for IPV (ABI/ABI-R) and controlling behaviors (CBS) for the 25 participants and 11 (ex)-partners who were interviewed at baseline and end of ADVANCE-D, were regrouped into three groups—where the scores increased, decreased or stayed the same from baseline to end of ADVANCE-D. Match paired t-tests and Wilcoxon signed-rank tests were performed for IPV outcomes pre and post program where normality could and could not be assumed, respectively. Interviews and focus groups (staff) were recorded and transcribed verbatim and patterns in themes across different participants and groups of participants were explored using the framework approach (89). Data for each time point interview and category of interviewee were merged into a single framework to enable comparison, interpretation, and synthesis of longitudinal data (90). Codes were developed and data were thematically coded. The COnsolidated criteria for REporting Qualitative research Checklist were followed during analysis (91).

## 4 Results of the multi-center study

### 4.1 Feasibility results

**Figure 3:**
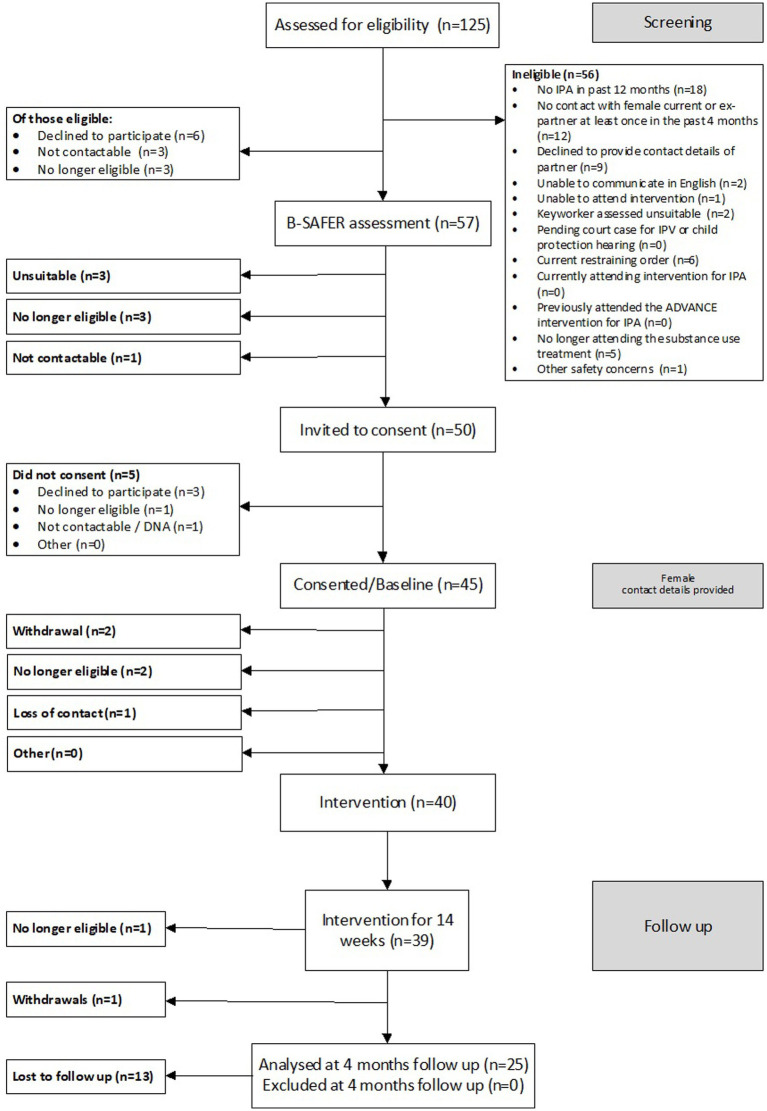
CONSORT diagram for male participants in the ADVANCE-D non-randomized feasibility study.

**Figure 4:**
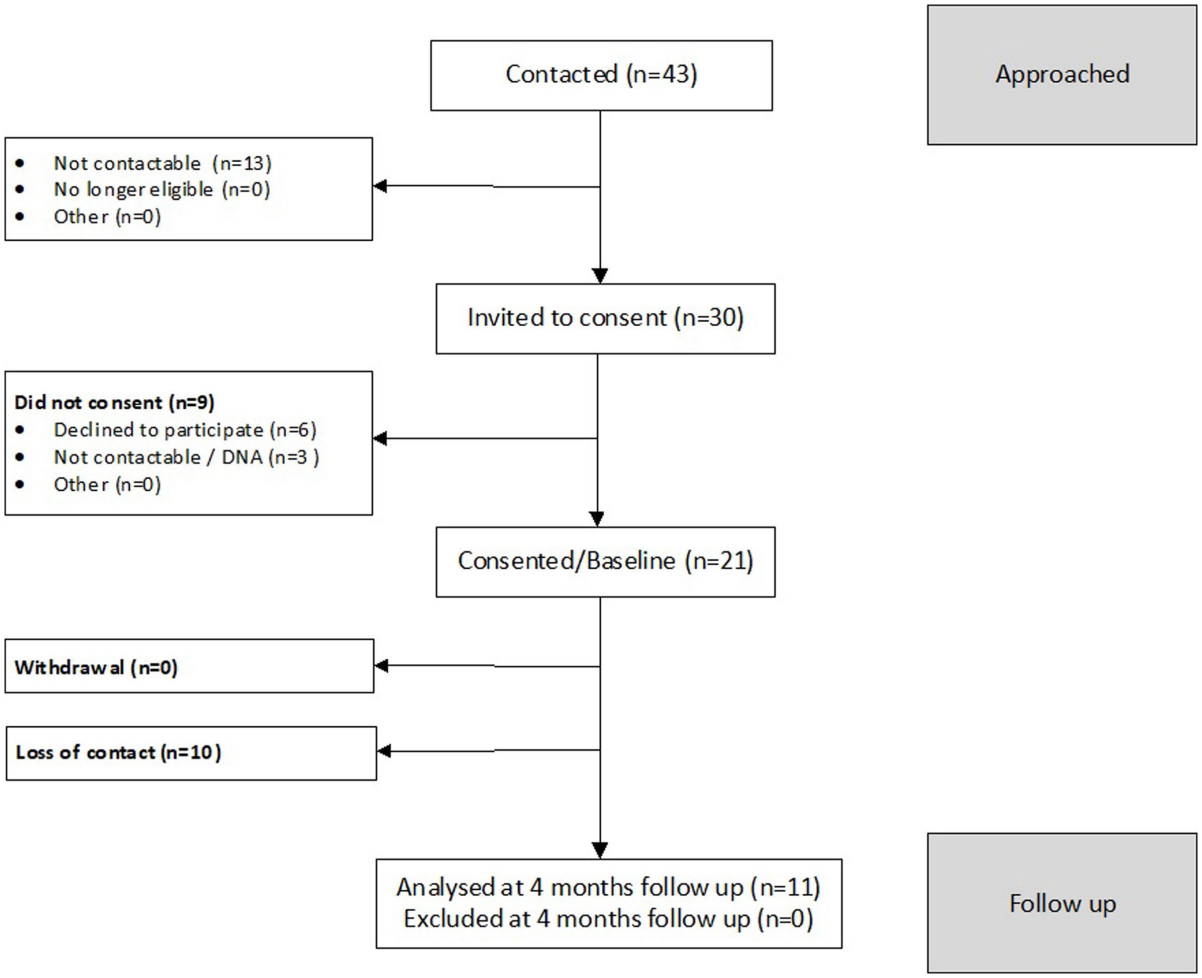
CONSORT diagram for female participants in the ADVANCE-D non-randomized feasibility study.

We determined the feasibility of delivering ADVANCE-D by assessing eligibility, recruitment, follow-up, adverse events, and uptake rates [of ADVANCE-D for participants and ISS for (ex)partners]. One hundred and twenty-five men were screened for eligibility, 69 of whom met criteria for inclusion in the study (55.2%). A B-SAFER risk assessment was completed with 57 of the 69 eligible men (82.6%), of whom 54 were suitable. Forty-five participants were recruited from seven community substance use treatment services in England (London *n* = 3, the West Midlands *n* = 1, and the South West *n* = 1), Wales (South Wales *n* = 1), and Scotland (Lothian *n* = 1). Twenty-one female current or ex-partners were recruited. Screening, recruitment, and follow-up of participants and their (ex)partners are described in more detail in Figures 3, 4. The reasons men were not eligible to participate are presented in Figure 3 and the reasons their (ex)partners did not participate in the research are detailed in Figure 4.

Table 1 presents the characteristics of male participants and their (ex)partners. Table 2 describes the feasibility parameters for participants and their (ex)partners. Of the 45 participants recruited, 22 were interviewed qualitatively at least once about their experiences of taking part in ADVANCE-D: four were interviewed once, 10 were interviewed twice, six were interviewed three times, and two were interviewed four times. Of the 21 (ex)partners recruited, 11 were interviewed qualitatively: eight were interviewed once, and three were interviewed twice. All 12 facilitators (three were interviewed once, eight were interviewed twice, and one was interviewed three times) and seven ISS workers (four were interviewed once, three were interviewed twice) were interviewed.

**Table 1:** Participant characteristics.

Characteristics		Men (*n* = 45)	Women (*n* = 21)
Age at consent	Mean (SD)	40.0 (8.5)	38.5 (9.7)
		*n* (%)	*n* (%)
Ethnic group	White	34 (75.6)	15 (71.4)
	Mixed/multiple backgrounds	4 (8.9)	4 (19.0)
	Asian	3 (6.7)	1 (4.8)
	African	3 (6.7)	0 (0.0)
	Any other ethnic group	1 (2.2)	1 (4.8)
Highest qualification	No formal qualifications	10 (22.2)	3 (14.3)
	High School qualifications	10 (22.2)	8 (38.1)
	Technical/vocational qualifications	17 (37.8)	4 (19.0)
	Degree or higher degree	4 (8.9)	2 (9.5)
	Other qualifications	4 (8.9)	4 (19.0)
Employment status	Employed	13 (28.9)	9 (42.9)
	Looking after home/family	1 (2.2)	2 (9.5)
	Unemployed and looking for work	10 (22.2)	1 (4.8)
	Unable to work due to long term sickness	19 (42.2)	9 (42.9)
	Retired from paid work	0 (0.0)	0 (0.0)
	In education	0 (0.0)	0 (0.0)
	Other	2 (4.4)	0 (0.0)
Sleeping arrangements	In a hostel or supported accommodation	1 (2.2)	1 (4.8)
	Sleeping on somebody’s sofa/floor	4 (8.9)	1 (4.8)
	Temporary accommodation	3 (6.7)	1 (4.8)
	Housed—in own tenancy	20 (44.4)	11 (52.4)
	Housed—in someone else’s tenancy	15 (33.3)	4 (19.0)
	Other	2 (4.4)	3 (14.3)
Relationship status with (ex)partner	Together and living together	24 (53.3)	10 (47.6)
	Together but living apart	8 (17.8)	7 (33.3)
	In the process of splitting up	1 (2.2)	0 (0.0)
	The relationship has ended and living apart with no contact	2 (4.4)	1 (4.8)
	The relationship has ended and we are living apart and still have contact	4 (8.9)	2 (9.5)
	Remain friends	2 (4.4)	0 (0.0)
	Be civil for sake of children	4 (8.9)	1 (4.8)
		mean (SD)	mean (SD)
Alcohol use disorders identification test score		16.2 (12.5)	6.4 (8.5)
Drug use disorders identification test score		20.1 (14.0)	7.1 (11.6)

**Table 2:** Feasibility estimates for male participants and (ex)-partners in the ADVANCE-D non-randomized feasibility study.

Male participants			Female (ex)-partners of men in the study		
Feasibility parameters	Proportion	Proportion %	Feasibility parameters	Proportion	Proportion %
Eligibility rate	69/125	55.2	-	-	-
Suitability rate	47/125	45.6	-	-	-
Recruitment rate	45/69	65.2	Recruitment rate	21/43^*^	48.8
ADVANCE-D uptake (attended at least one session)	39/40	86.7	ISS support uptake	13/15	86.7
Follow-up rate	25/45	55.6	Follow-up rate	11/21	52.4

^*^Only 43 (ex)partners were contacted as we were aware at that time that two men would not be not offered ADVANCE-D.

Forty participants were offered ADVANCE-D: 16 from London, nine from the South West, six from the West Midlands, five from Lothian and four from South Wales. Five of the 45 participants recruited were not offered ADVANCE-D as two were no longer eligible, two were withdrawn and contact was lost with one man prior to the program starting Six groups were delivered with an average of 6.8 men per group (range 4–9), one of which comprised participants from two different substance use treatment services (Lothian and South Wales). At the end of the program, a structured questionnaire was administered by researchers to 55.6% (25/45) of participants and 52.4% of their (ex)partners (11/21). Three severe adverse events were identified but found to be unrelated to the study.

### 4.2 Acceptability results

We determined acceptability by assessing attendance and completion rates for participants, therapeutic alliance and ratings for each website session, and multiple perspectives qualitative data.

Attendance varied by site and by type of session, with group sessions having the highest attendance (Table 3). On average, participants attended 11.4 (SD 9.1) of the 32 sessions (*n* = 40 participants). Of all sessions (goal setting session, “welcome” online preparation group session and six online group sessions, 12 website sessions and 12 coaching calls) offered, 46.8% (455/973) were attended (if participants had withdrawn/been discharged, or if the session was not delivered by staff, it was considered the session was not offered) (Table 3). One-month post-intervention, 30 of the 40 participants (75%) offered ADVANCE-D were invited to attend the refresher session; 11 (37%) of whom did so. The refresher session was not offered for the following reasons: participant withdrew (*n* = 1), participant no longer continuing with study intervention (*n* = 2), and site no longer delivering intervention (*n* = 7). While personal characteristics play a role in attendance, some contextual factors may have also had an impact. The delivery of ADVANCE-D progressed successfully in South West and Lothian/South Wales with good attendance by participants. In the West Midlands, two participants were thought to have sold their tablet prior to taking part and others found it difficult to fit attending the group around work, with one participant joining the group on his lunch break by phone and struggling to see videos. In all sites except the South West, the delivery of the six group sessions was interrupted by the Christmas break. In one of the three London sites, the second group was conducted in January. No participants joined the third group a fortnight later. This was then offered again but no one attended. The lead facilitator then went on leave for 2–3 weeks after which the group was offered again. When no one attended, it was decided to offer the remaining participant (who had been partially engaging via phone calls) an individual session but he failed to attend. One man in London site 1 had dropped out after reporting his tablet computer had been stolen. Although offered a new sim card, he proved uncontactable. In the West Midlands, due to the Christmas break and poor attendance, there was a five-week gap between online groups 2 and 3, instead of 2 weeks.

**Table 3:** Attendance at ADVANCE-D program by site.

Site	Core sessions (individual goal setting session + 7 groups)^*^		Website sessions^*^		Coaching calls^*^	
	*N*	Attended—*n* (%)	*N*	Attended—*n* (%)	*N*	Attended—*n* (%)
London 1	32	23 (71.9)	45	26 (57.8)	48	21 (43.8)
London 2	29	14 (48.3)	13	7 (53.8)	24	2 (8.3)
London 3	26	21 (80.8)	26	16 (61.5)	31	5 (16.1)
Lothian	39	29 (74.4)	60	33 (55.0)	60	29 (48.3)
South Wales	32	20 (62.5)	48	14 (29.2)	48	4 (8.3)
South West	65	56 (86.2)	91	52 (57.1)	93	57 (61.3)
West Midlands	44	18 (40.9)	55	2 (3.6)	64	6 (9.4)
Total	267	181 (67.8)	338	150 (44.4)	124	368 (33.7)

^*^Of sessions offered.

#### 4.2.1 Participants’ views on digitally-supported delivery

Many participants preferred the convenience of digitally-supported delivery stating accessing it from their homes and talking to facilitators by phone was easier *“makes it easier… it’s not as daunting*” than traveling into a service especially for those with social anxiety or who were working: *“It’s great that you have been able to run it online, because [I have] social anxiety. At the start of the course I would not really be up for face-to-face meetings, plus fears of COVID”* (ID 725). While several participants had felt *“anxious”* or *“nervous”* about attending the online group, they reported feeling able to open up, and appreciated listening to others doing the same, with participants articulating that this atmosphere felt non-judgmental, reduced shame and felt cathartic. Some stated they would have preferred an in-person group: *“I prefer face to face…you are a little bit more engaged”* (ID 401). Overall, participants reported good group dynamics. Most said it was *“good to hear other people in your position*” and that this made them feel they were *“not the only person in the situation”* (ID 206). Perceptions of group dynamics were linked to group size. In larger groups some participants found it difficult to interject finding it hard to know if they were talking too much as “*it’s a little bit harder to read the room ‘cos everyone’s* [*online*],” (ID 401). However, when there were few participants in groups, participants felt they were “*not getting any input* (from others)*”* (ID 131). The group that included participants from two different areas and services (South Wales/Lothian), was well received and participants liked the anonymity this allowed.

For some participants, setting up the tablet to access the online groups and website sessions proved difficult and frustrating, although participants reported receiving timely online technical support from a researcher who resolved issues quickly.

Participants appreciated the expertise of staff in facilitating groups, describing facilitators who made the group feel “safe” and that they felt included: *“They’ve been hosted really well. There were no disagreements between the guys…When talking about such a sensitive subject, you might expect more reactions and whatnot, but you guys obviously brought a vibe that meant that that wasn’t really a thing…which is good… it flowed really well. Making it a safe space.”* Table 4 demonstrates that at the end of ADVANCE-D participants scored therapeutic and working alliance high.

**Table 4:** Therapeutic and working alliance scores reported by participants at the end of ADVANCE-D.

Score	N	Mean (SD)	Median (IQR)
Working alliance inventory applied to virtual and augmented reality (WAI-VAR) score	22	64.7 (18.4)	69.5 (57.0–81.0)
California psychotherapy alliance scale (CALPAS) score	25	5.6 (1.0)	5.8 (4.8–6.2)

Participants were asked to rate each website session after completion from 1 (strongly disagree) to 7 (strongly agree). Overall, participants found the website easy to use (mean 5.8; SD 1.7); understood each session’s purpose (mean 6.2; SD 1.1); increased their knowledge and skills about topics covered in each session (mean 5.9; SD 1.3); and were able to concentrate (mean 5.8; SD 1.4). Participants were generally positive about the website: *“It was great. It was easy to use. I was impressed. It was better than I expected”* (ID 410) with *“very valid and great content”* (P415). They found the website sessions reinforced what they had learnt in the group sessions and vice versa and they appreciated the opportunity to work through them in their own time: *“When you do the session by yourself online after the group, I find that l helpful in a different way… you talk about stuff in the group and then you go and do the online session and then that’s what I sit down with the notebook and make notes. They work very well together”* (ID 411). While some participants thought having an online coach was *“better…to actually see a face, a person talking”* others felt that listening to him *“takes too long, the avatar talking. I just read through the thing and get it done*” (ID 402), *“I did not like listening to him talk to me so I had to mute him off all the time and read it myself”* (ID 415).

#### 4.2.2 Facilitators’ views on digitally-supported delivery

Most facilitators reported that issues with technology or poor Wi-Fi were disruptive to delivery of the online group. Despite technology issues affecting the ease with which people were able to engage with the groups, most facilitators reported good group dynamics; with the participants *“interact[ing] well”* (London site 1, Facilitator 1) with each other despite different personalities. In managing more difficult group dynamics, having a co-facilitator was viewed as key, because it enabled facilitators to *“take a breath”* (South Wales, Facilitator 1), during the group, *or “manage if someone was being disruptive or there was no talk”* (South West, Facilitator 1). In contrast, facilitators felt that delivering the group virtually might have led to groups being less *“bonded”* (London site 2, Facilitator 2 and South West, Facilitator 2), with less *“rapport”* between the participants and facilitators (West Midlands, Facilitator 2), however, these issues were helped by putting the first session aside *“for group cohesion”* (South Wales, Facilitator 1).

Delivering the group virtually was believed to make it more “accessible” (London site 3, Facilitator 1) and offered participants more *“flexibility […] to attend”* given work and other commitments (West Midlands, Facilitator 2), as well as being *“protective”* (London site 3, Facilitator 1), in giving them more space to reflect.

#### 4.2.3 ISS workers’ views on digitally-supported delivery

Integrated support service staff reported that (ex)partners who had requested calls were often difficult to contact and were not always in a private setting or alone when the ISS called. ISS staff found that some required a brief check-in call, while others required longer support calls: *“Of the small group there are about five* [(ex)partners to support]*, one is taking up a lot of the engagement, she finds it good to have space to talk and share her experiences. The rest it’s just checking in and the others have said they do not need support [from ISS] it’s just [a] check in, a 10-min call. Whereas the other one is like an hour, or if you do not stop her…2 h”* (London 1, ISS). There was variability in how much ISS staff reported looking at the ADVANCE-D website. While some reported finding the website useful, reinforcing existing knowledge and a good source of “refresher” messages, others reported not looking at the website or only looking at it once.

#### 4.2.4 Male participants’ views on ADVANCE-D program content

Overall, participants found the content *“really good”* (ID 726). Participants found the video clips *“helpful”* especially in highlighting *“what could he* [the perpetrator] *have done better*” (ID 205): *“[the videos] are good. It makes you, like I said, see things from other angles and stuff…It sticks in your head a bit more… easier to take in”* (ID 537). Most participants found the tools useful for meeting their goals: *“It’s given me the tools I need to do things a bit different, and stop things getting worse”* (ID 726). Structured time out was by far the most popular tool used with positive outcomes: *“Usually I would just storm off, grab money, just go take drugs, do whatever, but now I’ll actually sit down. I’ll think of the consequences of the actions before I do it. Not saying it stops me all the time doing it, but I think about what I’m going to do before I do it*… *It makes you actually think about everything, and about their side”* (ID 537). One participant mentioned when sending texts to an ex-partner, taking a time-out allowed him *“To take a step back, like, take a time out, whether that be just relax, take a deep breath. Think before you say…try and be a bit more reserved than just lashing out verbal abuse or written abuse. Just think, ‘No, I do not have to act like that’”* (ID 415).

Group sessions ended and website sessions started with a meditative breathing exercise to calm participants’ emotions and focus their attention. A few participants found them helpful and had started using them *“quite a lot”* (ID 308) to *“take a step back*… *once you start concentrating on your breathing, you calm down and then you could have a better approach to the way you wanna deal with things”* (ID 402).

Participants who received coaching calls appreciated the individual support provided, the opportunity for *“One-to-one was definitely, I would say, the best because you can open up more”* (ID 717), for follow-up and to ask questions about the website exercises and course content: *“Coaching calls were absolutely brilliant and when I was going through tough times, she’s [the facilitator] a really good listener. She’s really good at what she does. She was a great support. They reinforced a lot of the messages that we were doing in the groups, and it was real-time support with what was going on in my life, which I think is invaluable”* (ID 320).

#### 4.2.5 Facilitators’ views on ADVANCE-D program content

Facilitators thought the program content was *“valuable”* and *“comprehensive,”* however many found it *“unrealistic”* to cover the content in the time allocated for groups: *“Both [Facilitator 1] and I agree the content is fantastic. The level of time we are given each time is unrealistic. I do not think we can deliver all of that. But the content itself is really valuable. It is very useful. There is nothing I would want to expand further on. It was very comprehensive content”* (London site 3, Facilitator 2). While some facilitators were positive about the website content, particularly the videos which they described as “*really good”* (London 1, Facilitator 2), others described the look of the website and online coach as “*clunky”* (London 3, Facilitator 1). In addition, the challenge of getting *“[participants] to do it in the right order at the right time”* (London 2, Facilitator 2) was highlighted. Since the study the time scheduled for each group session has been increased and the platform for hosting ADVANCE-D has been changed, therefore the technical issues experienced should be reduced.

#### 4.2.6 Facilitators and ISS views on risk management

Facilitators valued the *“thorough”* risk assessment at the beginning of the program.

Four case management meetings took place during each cycle of ADVANCE-D between facilitators and ISS. Where facilitators were able to meet regularly with the ISS, the information sharing that this enabled was particularly valued: *“So, it was quite interesting because we got a lot of information from the partners that we had not been provided before, as we were, kind of, going through the (risk assessment) process and things like that. So, there was information that was coming to light from the partners themselves that was quite different to the information we were getting from the men”* (South Wales, Facilitator 1). ISS staff in this site gave similar reports of a useful case management meeting: *“So we discussed the case, and I think actually they learnt a lot from me. What my women were saying about their partners, that, partners might not have been really aware and – how high risk they were. So those meetings were really useful”* (South Wales, ISS). While sharing information about risk did not always take place in formal case management meetings, facilitators and ISS staff gave examples of information sharing by email (London site 1, West Midlands). Several facilitators reported insufficient or limited communication with the ISS (London site 3). However, where a change in risk was identified or any issues were “*flagged up*” these were discussed either individually or collectively between the ISS, facilitator and the ADVANCE-D clinical lead to ensure risk was appropriately managed.

### 4.3 Exploratory IPV outcomes

Baseline and follow-up measures of IPV perpetration and victimization are presented in Tables 5–7. Participants at baseline (*n* = 44) had a mean ABI perpetration score of 40.6 and a CBS-R perpetration score of 2.2. The subset of participants who remained active in the research and were assessed at follow-up (*n* = 25) had a mean ABI perpetration score of 34.2 and a mean CBS-R perpetration score of 0.8 (Table 5). Reductions in perpetration scores were reported by the 25 participants interviewed pre and post program for the following outcomes: ABI Perpetration (*p* < 0.05), Propensity for Abusiveness Scale (anger) (*p* < 0.05), Controlling Behaviors Scale (partial) (Perpetration) (*p* = 0.08), and using children against partner (*p* < 0.05) (Tables 6, 7).

**Table 5:** Client centered outcomes for men and their (ex)-partners’ in the ADVANCE-D non-randomized feasibility studyMeasure.

Measure	Baseline			Follow-up		
Male participants	*N*	Mean (SD)	Median (IQR)	*N*	Mean (SD)	Median (IQR)
Abusive behavior inventory (ABI) (perpetration) score	44	40.6 (8.9)	38.0 (35.0–46.5)	25	34.2 (6.1)	33.0 (29.0–38.0)
Controlling behaviors scale (partial) score	44	2.2 (2.1)	2.0 (0.0–3.5)	25	0.8 (1.3)	0.0 (0.0–1.0)
Use of social media in past 4 months score	44	2.8 (1.0)	2.0 (2.0–4.0)	25	2.2 (0.4)	2.0 (2.0–2.0)
Locked in in the past 4 months score	44	1.1 (0.4)	1.0 (1.0–1.0)	25	1.0 (0.2)	1.0 (1.0–1.0)
Stalking in past 4 months score	44	3.0 (1.4)	2.0 (2.0–4.0)	25	2.4 (0.9)	2.0 (2.0–2.0)
Using children against partner in past 4 months score	44	4.6 (3.1)	5.0 (3.0–6.0)	25	4.2 (2.2)	5.0 (5.0–5.0)
Propensity for abusiveness scale (anger)	44	36.2 (10.6)	37.5 (29.0–42.5)	25	30.6 (11.8)	27.0 (24.0–40.0)
(Ex)partners						
Revised abusive behavior inventory (ABI-R) (victimization) score	21	47.7 (18.7)	44.0 (34.0–52.0)	11	38.8 (15.5)	31.0 (27.0–51.0)
Controlling behaviors scale (partial) score	21	3.8 (4.0)	3.0 (1.0–5.0)	11	2.6 (3.0)	1.0 (0.0–5.0)
Use of social media in past 4 months score	21	3.2 (1.3)	3.0 (2.0–4.0)	11	2.5 (0.8)	2.0 (2.0–3.0)
Locked in in the past 4 months score	21	1.1 (0.3)	1.0 (1.0–1.0)	11	1.5 (1.2)	1.0 (1.0–1.0)
Stalking in past 4 months score	21	3.5 (1.5)	4.0 (2.0–4.0)	11	2.3 (0.6)	2.0 (2.0–2.0)
Using children against partner in past 4 months score	21	4.5 (2.7)	5.0 (5.0–6.0)	11	2.4 (2.5)	1.0 (0.0–5.0)

**Table 6:** Pre and post IPV perpetration and victimization outcomes for the 25 men and 11 (ex)-partners’ where normality is assumed.

	*N*	Mean difference	95% confidence interval	*p* value
*Participants*				
ABI—Perpetration	25	4.24	0.51–7.97	0.0277
Propensity for abusiveness scale (anger)	25	4.72	1.13–8.31	0.0122
*(Ex)partners*				
ABI—victimization	11	10.27	1.51–19.03	0.0259
Controlling behaviors scale (partial) (Victimization) score	11	1.55	−0.52-3.61	0.1268

**Table 7:** Pre and post IPV perpetration and victimization outcomes for the 25 men and 11 (ex)-partners’ where normality is not assumed.

Score	*N*	Positive difference (A decrease)	No difference	Negative difference (An increase)	*p* value
*Participants*					
Controlling behaviors scale (partial) (Perpetration)	25	10	11	4	0.0816
Use of social media	25	5	18	2	0.2050
Locked in	25	0	24	1	0.3173
Stalking	25	4	17	4	0.9608
Using children against partner	25	10	13	2	0.0141
*(Ex)partners*					
Use of social media—victimization	11	6	3	2	0.1025
Locked in—victimization	11	1	8	2	0.4899
Stalking—victimization	11	7	3	1	0.0201
Partner using children against her	11	6	5	0	0.0161

(Ex)partners at baseline (*n* = 21) had a mean ABI-R victimization score of 47.7 and a mean CBS-R victimization score of 3.8. The subset of (ex)partners who remained active in the research and were assessed at follow-up (*n* = 11) had a mean ABI victimization score of 38.8 and a CBS-R victimization score of 2.6 (Table 5).Reductions in victimization scores were reported by the 11 (ex)partners interviewed pre and post program for the following outcomes: ABI Victimization (*p* < 0.05), experiencing stalking behaviors (*p* < 0.05) and (ex)partner using children against her (*p* < 0.05) (Tables 6, 7). The process evaluation demonstrated that participants reported understanding the full range of abusive behaviors, particularly recognizing emotional and financial abuse; understanding the impact of IPV on their (ex)partners; and increasing respectful egalitarian communication, as opposed to aggressive or passive-aggressive communication. Many participants also reported examples of using their new skills to avoid being abusive. Many, but importantly not all participants and their (ex)partners reported positive behavior change in relation to the use or experience of IPV, respectively. These findings will be reported in detail elsewhere (92).

## 5 Discussion

Despite *“considerable concern about the use of ‘online’, ‘virtual’, or ‘digital’ programs delivered remotely”* (32), we found it was possible to adapt face-to-face content from our ADVANCE group program for digitally-supported delivery (consisting of online groups, self-completed website sessions and coaching calls) and feasible to deliver it remotely to participants receiving substance use treatment. To our knowledge this is the first evaluation of a digitally-supported perpetrator program (for men who use substances) (70, 93).

Similar to an exploratory study of an online court-mandated perpetrator program conducted pre-COVID in the United States (32), we found higher retention and attendance by participants in substance use treatment receiving ADVANCE-D remotely than our in-person ADVANCE group (26), although direct comparison with other perpetrator interventions for men who use substances, including the in-person ADVANCE, was not possible due to the heterogeneity in the duration and format of interventions. In the United States, 29 court-mandated perpetrators who used substances were allocated to a 12-session integrated individual intervention and 70% completed eight core sessions (94), compared to 68% in ADVANCE-D. In the Netherlands, 27 men attending IStop, a 16-session group perpetrator intervention for men in substance use treatment, reported that 44% completed at least 75% of sessions (defined as treatment completion), with a mean of 9/16 sessions attended (95). In ADVANCE-D, 59% of participants completed at least 75% of the eight core sessions and 47% of all 32 sessions offered were completed. Forty-four percent of ADVANCE-D website sessions offered were completed. This finding is similar to one study of 32 non-court-mandated, non-substance misusing perpetrators which found 44% completed all eight online modules and 75% completed at least half the modules (guided self-help delivered via the Internet with an identified therapist who provided support and guidance of therapeutic activities) (96). We have shown that ADVANCE-D has comparable and potentially higher engagement and retention than other community perpetrator interventions delivered in-person and online for men who use substances, including when compared to our in-person group delivery of ADVANCE. However, we do not know how the isolating factors of COVID restrictions impacted the engagement and acceptability of ADVANCE-D.

Only 55.6% of participants and 52.4% of their (ex)partners were followed-up. Attrition is a major challenge when evaluating the effectiveness of IPV perpetrator programs (97, 98). Establishing individualized participant retention plans using various strategies to maximize retention (e.g., via phone, email, through emergency and service provider contacts) and ensuring researchers build rapport and maintain relationships for the duration of the study (e.g., same researcher completes all contacts and interviews where possible), may help to increase retention in longitudinal research with hard-to-reach participants (99). Substance use is a significant predictor of drop-out (14, 17, 100), with one trial reporting higher drop-out rates among men with alcohol abuse problems (36%) than those without (23%) (101). Research suggests around 20–80% of perpetrators drop out of programs (17, 98, 102, 103), however there is a lack of standard definition of program attrition, making cross-study comparison problematic (104). Richards et al. (104) found that 26% of perpetrators dropped out following intake assessment and did not complete any program sessions, while a further 26% completed some program sessions prior to dropping out. They found early non-engagers were more likely to have mental health problems and engagers who then dropped out were more like to have substance use problems. Presenting findings on attrition separately for early non engagement, no shows at the first session and for those who engage then drop-out (104), may facilitate a better understanding of who the program works best for and where extra support may be needed. In our study, five men who consented did not take up the intervention: two men decided they no longer wanted to take part (with no reason given), two no longer met the inclusion criteria and one was non-contactable to complete the baseline interview. During the program delivery, four men were discharged from the substance use treatment service: two chose to discharge themselves (one of whom attended none of the intervention) and two were discharged for non-engagement with the substance use treatment service (both had attended two ADVANCE-D group sessions). While we did seek to record reasons for program non-engagement and drop-out, this was not always possible as men were not always contactable. Future studies should use administrative data (e.g., health and social care records, police and prison data) to measure program outcomes in the short, medium and long term to address the issue of attrition and attempt qualitative follow-up with those who drop out or do not engage (17). Adequately powered studies should explore whether the type and severity of IPV perpetrated or experienced predicted non-engagement and attrition in the research. Given our small sample size, it was not possible to meaningfully explore predictors of drop-out. Our study took place during Covid-19 restrictions which may have resulted in more illness among staff and clients, as well as greater pressure on staff alongside delivering ADVANCE-D which impacted the continuity of program delivery. The timing of program delivery in our study may also have contributed to non-engagement as sessions over the holiday period were delayed or not delivered to schedule. To improve continuity of program delivery, future studies should ensure the program is not scheduled to be delivered over the holiday period to try to retain men in the program. Moreover, ensuring an adequate number of facilitators are trained would avoid cancelation of sessions when facilitators are on annual leave, are sick or leave their position.

The differences in perpetration (and victimization) scores pre and post program show that some men who were followed-up had reduced their use of abusive behaviors. However, given the small sample size and non-randomized study design, we do not know if these differences are due to ADVANCE-D, time, participant factors, or chance. Moreover, the lower ABI/ABI-R mean scores at follow-up could be due to attrition by participants with high scores at baseline. Engagement and retention, while higher than other perpetrator programs for men in substance use treatment, was still fairly low overall with one group ceasing to continue due to dropouts and non-attendance. Due to study constraints, long term follow up was not possible, but is required to explore whether these findings are maintained and whether ADVANCE-D is more effective than usual treatment. More research is needed before conclusions can be made about the efficacy of ADVANCE-D. An RCT is planned for ADVANCE-D with men in probation throughout the United Kingdom which will address these concerns.

Overall, the intervention content was well-received by men and facilitators. Some men preferred digital over in-person sessions as they offered increased accessibility.

Since the feasibility study was conducted, we have twice updated the usability, look and feel of the website sessions to address the feedback provided, and to provide users with options for online coaches. Little research has explored user preferences for an online therapist/avatar’s portrayed gender and ethnicity outside of the gaming context. Where we could find evidence in health or social care research, most users chose the male and female avatar that portrayed their own gender and ethnicity (105, 106). Research also found that users welcomed diverse avatars and animations (107). Disclosure of sensitive information (including drug use, sexual abuse and domestic violence victimization) was more likely when avatars appeared similar in age to participants (108). Following the feasibility study, developers have created three new avatars portraying White, Black, and South Asian ethnicities, which were selected and tested with 16 people with lived experience. The ADVANCE-D manual and website sessions will also be available in Polish, Urdu and Panjabi.

The pandemic necessitated a move from face-to-face delivery of interventions to online delivery (35, 109, 110). For online interventions, it is important that the digital literacy and digital poverty of service users is addressed to ensure the ability to engage in ADVANCE-D (64–66). While there is no review of the efficacy of online delivery of perpetrator programs during the pandemic, a review compared substance use treatment delivered using telehealth with in-person treatment during the pandemic and concluded that telehealth treatment was effective but not more effective than in-person treatment in terms of retention, therapeutic alliance, and substance use (111). A recent study with men in behavior change programs concluded that *“Websites or apps can provide a safe, private space for men to reflect on their behavior and its consequences; however, the lack of interpersonal interaction can make it challenging to balance non-judgmental engagement with accountability”* (55). ADVANCE-D addressed this concern by including coaching calls with a facilitator post-completion of each website session. However, staff need protected time to prepare, deliver and debrief after delivering group sessions and coaching calls. Supervised completion of ADVANCE-D website sessions and in-person (rather than remote) group and coaching sessions at substance use treatment services could enhance attendance, completion and engagement, and ensure adherence to all aspects of the intervention.

Integrated support for (ex)partners alongside regular case management meetings and clear and respectful information sharing protocols, are essential components of the ADVANCE and ADVANCE-D interventions. Co-training and integrity support for facilitators and ISS services is needed to build strong professional relationships across services working with participants and supporting (ex)partners.

## 6 Conclusion

Given that men who use substances are underserved in perpetrator programs and are also most likely to drop out of standard perpetrator programs (14, 23, 24), alternative approaches that address their specific needs and risks, such as ADVANCE-D are required. We were able to adapt the ADVANCE face-to-face group program to reduce IPV by men in substance use treatment for blended digitally-supported delivery based on available evidence of best practice with input from key stakeholders and people with lived experience. The results of the feasibility study showed that it was feasible to recruit, engage and follow up participants from substance use treatment to the ADVANCE-D program with enhanced risk management practices in place for (ex)partners, and exploratory outcomes are promising. An efficacy trial of ADVANCE-D is warranted with longer-term follow-up recommended. ADVANCE-D has long-term applicability post pandemic, including in other settings. Applicability of ADVANCE-D in other settings and populations remains to be tested.

## Data availability statement

The datasets presented in this article are not readily available because all data requests should be submitted to the corresponding author for consideration. Access to anonymized data may be granted following review. Requests to access the datasets should be directed to gail.gilchrist@kcl.ac.uk.

## Ethics statement

Ethics approval was granted by Yorkshire and The Humber-Sheffield Research Ethics Committee on January 25, 2021 (Reference: 19/YH/0445). The studies were conducted in accordance with the local legislation and institutional requirements. The participants provided their informed consent to participate in this study.

## Author contributions

GG was the chief investigator for the research program. EG led the adaptation of the ADVANCE Program, with support from AJ, KT, CiB, and GG. GG led the development of the study protocol and applied for ethical approval. GG, SD, AJ, JH, PR, GD, RT, KT, CPB, BL, ZZ, CaB, BC, SP, JL, CE, CiB, GF, and EG participated in the design of the study including the selection of outcome measures for the study and the recruitment procedures. GG. SD, JH, AJ, KT, GD, RT, and CPB conducted scoping reviews to provide evidence for adaptation and recruited and interviewed participants. CaB led the website design and development. BC and ZZ conducted the statistical analysis. SP and JL conducted the economic evaluation. PR and SD led the process evaluation methodology with input and analysis by JH, BL, AJ, KT, GD, RT, and CPB. GG drafted the manuscript with support from all authors who approved the final manuscript prior to submission.

## References

[1] World Health Organization (2012). Understanding and addressing violence against women: intimate partner violence. Geneva: World Health Organization. Available at: https://apps.who.int/iris/bitstream/handle/10665/77432/WHO_RHR_12.36_eng.pdf?sequence=1&isAllowed=y (Accessed June 12, 2023)

[2] Liu M Cai X Hao G Li W Chen Q Chen Y. Prevalence of intimate partner violence among men who have sex with men: an updated systematic review and Meta-analysis. Sex Med. (2021) 9:10043. doi: 10.1016/j.esxm.2021.100433, PMID: 34571326 PMC8766270

[3] Peitzmeier SM Mannat M Kattari SK Marrow E Stephenson R Agénor A. Intimate partner violence in transgender populations: systematic review and meta-analysis of prevalence and correlates. Am J Public Health. (2020) 110:e1–e14. doi: 10.2105/AJPH.2020.305774, PMID: 32673114 PMC7427218

[4] Badenes-Ribera L Bonilla-Campos A Frias-Navarro D Pons-Salvador G Monterde-I-Bort H. Intimate partner violence in self-identified lesbians: a systematic review of its prevalence and correlates. Trauma Violence Abuse. (2016) 17:284–97. doi: 10.1177/1524838015584363, PMID: 26018210

[5] Chermack ST Murray RL Walton MA Booth BA Wryobeck J Blow FC. Partner aggression among men and women in substance use disorder treatment: correlates of psychological and physical aggression and injury. Drug Alcohol Depend. (2008) 98:35–44. doi: 10.1016/j.drugalcdep.2008.04.010, PMID: 18554825 PMC3771635

[6] World Health Organization (2013). Global and regional estimates of violence against women: prevalence and health effects of intimate partner violence and nonpartner sexual violence. Geneva: World Health Organization. Available at: https://www.who.int/publications/i/item/9789241564625 (Accessed June 28, 2023)

[7] Kim B Merlo AV. Domestic homicide: a synthesis of systematic review evidence. Trauma Violence Abuse. (2023) 24:776–93. doi: 10.1177/15248380211043812, PMID: 34510978

[8] Costa BM Kaestle CE Walker A Curtis A Day A Toumbourou JW. Longitudinal predictors of domestic violence perpetration and victimization: a systematic review. Aggress Violent Behav. (2015) 24:261–72. doi: 10.1016/j.avb.2015.06.001

[9] Gibbs A Dunkle K Ramsoomar L Willan S Jama Shai N Chatterji S. New learnings on drivers of men's physical and/or sexual violence against their female partners, and women's experiences of this, and the implications for prevention interventions. Glob Health Action. (2020) 13:1739845. doi: 10.1080/16549716.2020.1739845, PMID: 32202227 PMC7144308

[10] Choenni V Hammink A Van de Mheen D. Association between substance use and the perpetration of family violence in industrialized countries. A systematic review. Trauma Violence Abuse. (2017) 18:37–50. doi: 10.1177/1524838015589253, PMID: 26296740

[11] Oram S Trevillion K Khalifeh H Feder G Howard LM. Systematic review and meta-analysis of psychiatric disorder and the perpetration of partner violence. Epidemiol Psychiatr Sci. (2014) 23:361–76. doi: 10.1017/S2045796013000450, PMID: 23962668 PMC7192171

[12] Cafferky BM Mendez M Anderson JR Stith SM. Substance use and intimate partner violence: a meta-analytic review. Psychol Violence. (2018) 8:110–31. doi: 10.1037/vio0000074

[13] Gilchrist G Radcliffe P Noto A Flavia A. Prevalence and risk factors for IPV among males attending substance misuse treatment in England and Brazil. Drug Alcohol Rev. (2017) 36:34–51. doi: 10.1111/dar.12436 27709693

[14] Expósito-Álvareza C Santirso FA Gilchrist G Gracia E Lila M. Participants in court-mandated intervention programs for intimate partner violence perpetrators with substance use problems: a systematic review of specific risk factors. Psychosoc Interv. (2023) 32:89–108. doi: 10.5093/pi2023a7, PMID: 37383646 PMC10294470

[15] Smith PH Homish GG Leonard KE Cornelius JR. Intimate partner violence and specific substance use disorders: findings from the national epidemiologic survey on alcohol and related conditions. Psychol Addict Behav. (2012) 26:236–45. doi: 10.1037/a0028217, PMID: 21823768 PMC3883081

[16] Fulu E Jewkes R Roselli T Garcia-Moreno C. Prevalence of and factors associated with male perpetration of intimate partner violence: findings from the UN multi-country cross-sectional study on men and violence in Asia and the pacific. Lancet Glob Health. (2013) 1:187–207. doi: 10.1016/S2214-109 25104345

[17] Travers Á McDonagh T Cunningham T Armour C Hansen M. The effectiveness of interventions to prevent recidivism in perpetrators of intimate partner violence: a systematic review and meta-analysis. Clin Psychol Rev. (2021) 84. doi: 10.1016/j.cpr.2021.101974, PMID: 33497921

[18] Butters RP Droubay BA Seawright JL Tollefson DR Lundahl B Whitaker L. Intimate partner violence perpetrator treatment: tailoring interventions to individual needs. Clin Soc Work J. (2021) 49:391–404. doi: 10.1007/s10615-020-00763-y

[19] Stephens-Lewis D Johnson A Huntley A Gilchrist E McMurran M Henderson J. Interventions to reduce intimate partner violence perpetration by men who use substances: a systematic review and meta-analysis of efficacy. Trauma Violence Abuse. (2021) 22:1262–78. doi: 10.1177/1524838019882357, PMID: 31711372 PMC8649458

[20] Karakurt G Koç E Çetinsaya EE Ayluçtarhan Z Bolen S. Meta-analysis and systematic review for the treatment of perpetrators of intimate partner violence. Neurosci Biobehav Rev. (2019) 105:220–30. doi: 10.1016/j.neubiorev.2019.08.006, PMID: 31415863 PMC6742529

[21] Tarzia L Forsdike K Feder G Hegarty K. Interventions in health settings for male perpetrators or victims of intimate partner violence. Trauma Violence Abuse. (2020) 21:123–37. doi: 10.1177/1524838017744772 29333972

[22] Hashimoto N Radcliffe P Gilchrist G. Help-seeking behaviors for intimate partner violence perpetration by men receiving substance use treatment: a mixed-methods secondary analysis. J Interpers Viol. (2018) 36:3142–67. doi: 10.1177/0886260518770645, PMID: 29756559

[23] Timko C Valenstein H Lin PY Moos RH Stuart GL Cronkite RC. Addressing substance abuse and violence in substance use disorder treatment and batterer intervention programs. Subst Abuse Treat Prev Policy. (2012) 7:37. doi: 10.1186/1747-597X-7-37, PMID: 22958624 PMC3489609

[24] Rothman E Butchart A Cerda M (2003). Intervening with perpetrators of intimate partner violence: a global perspective. Geneva, Switzerland: World Health Organization. Available at: http://apps.who.int/iris/bitstream/handle/10665/42647/9241590491.pdf;jsessionid=96E444F7CCE506E302C041CB448F4264?sequence=1 (Accessed June 12, 2023).

[25] Gilchrist E Johnson A McMurran M Stephens-Lewis D Kirkpatrick S Gardner B. Using the behaviour change wheel to design an intervention for partner abusive men in drug and alcohol treatment. Pilot Feasibility Stud. (2021) 7:191–1. doi: 10.1186/s40814-021-00911-2, PMID: 34711276 PMC8551949

[26] Gilchrist G Potts L Radcliffe P Halliwell G Dheensa S Henderson J. Findings from a feasibility trial of the ADVANCE integrated intervention to address both substance use and intimate partner abuse perpetration by men in substance use treatment. BMC Public Health. (2021) 21:980. doi: 10.1186/s12889-021-11012-3, PMID: 34034690 PMC8147906

[27] Dheensa S Halliwell G Johnson A Henderson J Love B Radcliffe P. Perspectives on motivation and change in an intervention for men who use substances and perpetrate intimate partner abuse: findings from a qualitative evaluation of the Advance intervention. J Interpers Violence. (2022) 37:NP13342–72. doi: 10.1177/0886260521997436, PMID: 33715489 PMC9326801

[28] Radcliffe P Gadd D Henderson J Love B Stephens-Lewis D Johnson A. What role does substance use play in intimate partner violence? A narrative analysis of in-depth interviews with men in substance use treatment and their current or former female partner. J Interpers Viol. (2021) 36:10285–313. doi: 10.1177/0886260519879259, PMID: 31578902 PMC8581707

[29] Gadd D Radcliffe P Henderson J Stephens-Lewis D Johnson A Gilchrist G. The dynamics of domestic abuse and drug and alcohol dependency. Br J Criminol. (2019) 59:1035–53. doi: 10.1093/bjc/azz011

[30] Gilchrist G Dennis F Radcliffe P Henderson J Howard LM Gadd D. The interplay between substance use and intimate partner violence perpetration: a meta-ethnography. Int J Drug Policy. (2019) 65:8–23. doi: 10.1016/j.drugpo.2018.12.009, PMID: 30580114

[31] Gilchrist E Johnson A Thomson K Stephens-Lewis D Henderson J Gadd D. Substance use and intimate partner abuse (IPA): a descriptive model of the pathways between substance use and IPA perpetration for men. J Fam Violence. (2022) 38:855–68. doi: 10.1007/s10896-022-00395-5

[32] Bellini R Westmarland N. A problem solved is a problem created: the opportunities and challenges associated with an online domestic violence perpetrator programme. J Gender Viol. (2021) 5:499–515. doi: 10.1332/239868021X16171870951258

[33] Respect (2020). Responding to the challenges of COVID-19: Safeguarding and practice guidance for practitioners working with domestic abuse perpetrators. Version date: 31st 2020. Available at: https://hubble-live-assets.s3.amazonaws.com/respect/redactor2_assets/files/81/Respect_Covid19_Guidance_for_DA_Practitioners_March_2020.pdf (Accessed June 23, 2023).

[34] Work with Perpetrators—European Network (2023). Guidelines to ensure responsible perpetrator work during COVID-19. Available at: https://www.work-with-perpetrators.eu/covid-19 (Accessed June 23, 2023).

[35] Bellini R Westmarland N. “We adapted because we had to”: how domestic violence perpetrator programmes adapted to work under COVID-19 in the UK, the USA and Australia. J Aggress. (2023) 15:205–15. doi: 10.1108/JACPR-05-2022-0716

[36] Kourti A Stavridou A Panagouli E Psaltopoulou T Spiliopoulou C Tsolia M. Domestic violence during the COVID-19 pandemic: a systematic review. Trauma Violence Abuse. (2023) 24:719–45. doi: 10.1177/15248380211038690, PMID: 34402325 PMC10011925

[37] Gilchrist G Potts LC Connolly DJ Winstock A Barratt M Ferris J. Experience and perpetration of intimate partner violence and abuse by gender of respondent and their current partner before and during COVID-19 restrictions in 2020: a cross-sectional study in 13 countries. BMC Public Health. (2023) 23:316. doi: 10.1186/s12889-022-14635-2, PMID: 36782157 PMC9924203

[38] Peitzmeier SM Fedina L Ashwell L Herrenkohl TI Tolman R. Increases in intimate partner violence during COVID-19: prevalence and correlates. J Interpers Violence. (2022) 37:NP20482–512. doi: 10.1177/08862605211052586, PMID: 34866451 PMC9014340

[39] Roberts A Rogers J Mason R Siriwardena AN Hogue T Whitley GA. Alcohol and other substance use during the COVID-19 pandemic: a systematic review. Drug Alcohol Depend. (2021) 229:109150. doi: 10.1016/j.drugalcdep.2021.109150, PMID: 34749198 PMC8559994

[40] Schmidt RA Genois R Jin J Vigo D Rehm J Rush B. The early impact of COVID-19 on the incidence, prevalence, and severity of alcohol use and other drugs: a systematic review. Drug Alcohol Depend. (2021) 228:109065. doi: 10.1016/j.drugalcdep.2021, PMID: 34600257 PMC8455354

[41] Yardley L Ainsworth B Arden-Close E Muller I. The person-based approach to enhancing the acceptability and feasibility of interventions. Pilot Feasibility Stud. (2015) 1:37. doi: 10.1186/s40814-015-0033-z, PMID: 27965815 PMC5153673

[42] Yardley L Morrison L Bradbury K Muller I. The person-based approach to intervention development: application to digital health-related behavior change interventions. J Med Internet Res. (2015) 17:e30. doi: 10.2196/jmir.4055, PMID: 25639757 PMC4327440

[43] Whittaker R Merry S Dorey E Maddison R. A development and evaluation process for mHealth interventions: examples from New Zealand. J Health Commun. (2012) 17:11–21. doi: 10.1080/10810730.2011.649103, PMID: 22548594

[44] Manera K Hanson CS Gutman T Tong A. Consensus methods: nominal group technique In: Liamputtong P, editor. Handbook of Research Methods in Health Social Sciences. Singapore: Springer (2019)

[45] Stawarz K Preist C Tallon D Wiles N Kessler D Turner K. Design considerations for the integrated delivery of cognitive behavioral therapy for depression: user-centered design study. JMIR Ment Health. (2020) 7:e15972. doi: 10.2196/15972, PMID: 32880580 PMC7499168

[46] Bradbury K Watts S Arden-Close E Yardley L Lewith G. Developing digital interventions: a methodological guide. Evid Complement Altern Med. (2014) 2014:561320. doi: 10.1155/2014/561320, PMID: 24648848 PMC3932254

[47] Karyotaki E Efthimiou O Miguel C Bermpohl FMG Furukawa TA Cuijpers P. Internet-based cognitive behavioral therapy for depression: a systematic review and individual patient data network meta-analysis. JAMA Psychiatry. (2021) 78:361–71. doi: 10.1001/jamapsychiatry.2020.4364, PMID: 33471111 PMC8027916

[48] Sørensen HR Lillevoll KR Griffiths KM Wilsgaard T Eisemann M Waterloo K. The clinical effectiveness of web-based cognitive behavioral therapy with face-to-face therapist support for depressed primary care patients: randomized controlled trial. J Med Internet Res. (2013) 15:e153. doi: 10.2196/jmir.2714, PMID: 23916965 PMC3742404

[49] Mathiasen K Andersen TE Lichtenstein MB Ehlers LH Riper H Kleiboer A. The clinical effectiveness of blended cognitive behavioral therapy compared with face-to-face cognitive behavioral therapy for adult depression: randomized controlled noninferiority trial. J Med Internet Res. (2022) 24:e36577. doi: 10.2196/36577, PMID: 36069798 PMC9543221

[50] Sourander A McGrath PJ Ristkari T Cunningham C Huttunen J Lingley-Pottie P. Internet-assisted parent training intervention for disruptive behavior in 4-year-old children: a randomized clinical trial. Arch Gen Psychiatry. (2016) 73:378–87. doi: 10.1001/jamapsychiatry.2015.3411, PMID: 26913614

[51] Day JJ Sanders MR. Do parents benefit from help when completing a self-guided parenting program online? A randomized controlled trial comparing triple P online with and without telephone support. Behav Ther. (2018) 49:1020–38. doi: 10.1016/j.beth.2018.03.002, PMID: 30316482

[52] Vangrunderbeek A Raveel A Matheï C Claeys H Aertgeerts B Bekkering G. Effectiveness of guided and unguided online alcohol help: a real-life study. Internet Interv. (2022) 28:100523. doi: 10.1016/j.invent.2022.100523, PMID: 35330980 PMC8938279

[53] Martin J McBride T Masterman T Pote DI Mokhtar DN Oprea E (2020). Covid-19 and early intervention: evidence, challenges and risks relating to virtual and digital delivery. Early Intervention Foundation. Available at: https://www.eif.org.uk/report/covid-19-and-early-intervention-evidence-challenges-and-risks-relating-to-virtual-and-digital-delivery. (Accessed June 29, 2023)

[54] Taylor GMJ Dalili MN Semwal M Civljak M Sheikh A Car J. Internet-based interventions for smoking cessation. Cochrane Database Syst Rev. (2017) 2017:CD007078. doi: 10.1002/14651858.CD007078.pub5, PMID: 28869775 PMC6703145

[55] Tarzia L McKenzie M Addison MJ Hameed MA Hegarty K. “Help me realize what I’m becoming”: men’s views on digital interventions as a way to promote early help-seeking for use of violence in relationships. J Interpers Violence. (2023) 38:8016–41. doi: 10.1177/08862605231153885, PMID: 36762522 PMC10326356

[56] Morris J Bans MK. Developing digitally enabled interventions for prison and probation settings: a review. J Forens Pract. (2018) 20:134–40. doi: 10.1108/JFP-08-2017-0030

[57] Rübsamen N Akmatov MK Castell S Karch A Mikalojczyk RT. Factors associated with attrition in a longitudinal online study: results from the HaBIDS panel. BMC Med Res Methodol. (2017) 17:132. doi: 10.1186/s12874-017-0408-3, PMID: 28859617 PMC5580321

[58] Linardon J Fuller-Tyszkiewicz M. Attrition and adherence in smartphone-delivered interventions for mental health problems: a systematic and meta-analytic review. J Consult Clin Psychol. (2020) 88:1–13. doi: 10.1037/ccp0000459, PMID: 31697093

[59] Easton CJ Berbary CM Crane CA. Avatar and technology assisted platforms in the treatment of co-occurring addiction and IPV among male offenders. Adv Dual Diagn. (2018) 11:126–34. doi: 10.1108/ADD-03-2018-0003

[60] Hsu CW (2021). Eliciting information from adults: quality, quantity, and their willingness to disclose to an avatar interviewer. Doctoral dissertation. University of Otago. Available at: http://hdl.handle.net/10523/10775 (Accessed June 29, 2023).

[61] Clark RC Mayer RE. Applying the modality principle In: Taff R, editor. E-Learning and the Science of Instruction. San Francisco, CA: Wiley (2011). 115–30.

[62] Mathieu E Barratt A Carter SM Jamtvedt G. Internet trials: participant experiences and perspectives. BMC Med Res Methodol. (2012) 12:162. doi: 10.1186/1471-2288-12-162, PMID: 23092116 PMC3533967

[63] Todkill D Powell J. Participant experiences of an internet-based intervention and randomised control trial: interview study. BMC Public Health. (2013) 13. doi: 10.1186/1471-2458-13-1017, PMID: 24165325 PMC3871009

[64] Milward J Day E Wadsworth E Strang J Lynskey M. Mobile phone ownership, usage and readiness to use by patients in drug treatment. Drug Alcohol Depend. (2015) 146:111–5. doi: 10.1016/j.drugalcdep.2014.11.001, PMID: 25468818

[65] Nesvåg S McKay JR. Feasibility and effects of digital interventions to support people in recovery from substance use disorders: systematic review. J Med Internet Res. (2018) 20:e255. doi: 10.2196/jmir.9873, PMID: 30139724 PMC6127498

[66] Baier AL Kline AC Feeny NC. Therapeutic alliance as a mediator of change: a systematic review and evaluation of research. Clin Psychol Rev. (2020) 82:101921. doi: 10.1016/j.cpr.2020.101921, PMID: 33069096

[67] Weinberg H. Obstacles, challenges, and benefits of online group psychotherapy. Am J Psychother. (2021) 74:83–8. doi: 10.1176/appi.psychotherapy.20200034, PMID: 33525914

[68] Kivlighan DM Aloe AM Adams MC Garrison YL Obrecht A Ho YCS. Does the group in group psychotherapy matter? A meta-analysis of the intraclass correlation coefficient in group treatment research. J Consult Clin Psychol. (2020) 88:322–37. doi: 10.1037/ccp0000474 31855036

[69] Stewart L Usher A. A review of optimal group size and modularisation or continuous entry format for program delivery. Correctional service Canada. Research report, number R-215. (2009). Available at: https://www.csc-scc.gc.ca/005/008/092/005008-0215-01-eng.pdf (Accessed June 30, 2023).

[70] Vlais R Campbell E (2020). Alternative delivery formats for domestic and family violence perpetrator programs in the COVID -19 situation, RMIT Centre for innovative justice. Available at: https://www.doj.state.or.us/wp-content/uploads/2020/10/Alternative_delivery_formats-DV_perpetrator_programs-in_COVID-19.pdf (Accessed June 24, 2023).

[71] Richards L (2009). Domestic abuse, stalking and harassment and honour-based violence risk identification and assessment and management model. Available at: https://www.dashriskchecklist.co.uk/wp-content/uploads/2021/12/DASH-2009.pdf (Accessed June 29, 2023)

[72] Kropp PR Hart SD Belfrage H. (2010). Brief spousal assault form for the evaluation of risk (B-SAFER). Version 2. User manual. Vancouver: Proactive Resolutions.

[73] Inzlicht M Werner KM Briskin JL Roberts BW. Integrating models of self-regulation. Annu Rev Psychol. (2021) 72:319–45. doi: 10.1146/annurev-psych-061020-105721 33017559

[74] Pfund RA Ginley MK Rash CJ Zajac K. Contingency management for treatment attendance: a meta-analysis. J Subst Abus Treat. (2022) 133:108556. doi: 10.1016/j.jsat.2021.108556, PMID: 34210566 PMC8702584

[75] Ward T Brown M. The good lives model and conceptual issues in offender rehabilitation. Psychol Crime Law. (2004) 10:243–57. doi: 10.1080/10683160410001662744

[76] Gilchrist G Landau S Dheensa S Henderson J Johnson A Love B. The feasibility of delivering the ADVANCE digital intervention to reduce intimate partner abuse by men receiving substance use treatment: protocol for a non-randomised multi-Centre feasibility study and embedded process evaluation. Pilot Feasibility Stud. (2022) 8:163. doi: 10.1186/s40814-022-01116-x, PMID: 35907900 PMC9338654

[77] Sim J Lewis M. The size of a pilot study for a clinical trial should be calculated in relation to considerations of precision and efficiency. J Clin Epidemiol. (2012) 65:301–8. doi: 10.1016/j.jclinepi.2011.07.011 22169081

[78] Shepard MF Campbell JA. The abusive behavior inventory: a measure of psychological and physical abuse. J Interpers Viol. (1992) 7:291–305. doi: 10.1177/088626092007003001

[79] The Respect Standard (2022). 4th edition. Respect. Available at: https://hubble-live-assets.s3.amazonaws.com/respect/file_asset/file/1458/Respect_Standard_4th_edition_2022.pdf (Accessed June 28, 2023).

[80] Standards for Domestic Abuse Perpetrator Interventions (2023). Home office. Available online at: https://www.gov.uk/government/publications/standards-for-domestic-abuse-perpetrator-interventions/standards-for-domestic-abuse-perpetrator-interventions-accessible (Accessed June 28, 2023).

[81] Love B Henderson J Johnson A Stephens-Lewis D Gadd D Radcliffe P. The challenges of conducting qualitative research on “couples” in abusive intimate partner relationships involving substance use. Qual Health Res. (2021) 31:767–77. doi: 10.1177/1049732320975722, PMID: 33292083 PMC7885088

[82] Campbell J Webster D Glass N. The danger assessment: validation of a lethality risk assessment instrument for intimate partner femicide. J Interpers Violence. (2009) 24:653. doi: 10.1177/0886260508317180, PMID: 18667689 PMC7878014

[83] Postmus JL Stylianou AM McMahon S. The abusive behavior inventory–revised. J Interpers Violence. (2016) 31:2867–88. doi: 10.1177/0886260515581882 25900914

[84] Graham-Kevan N Archer J. Investigating three explanations of women’s relationship aggression. Psychol Women Q. (2005) 29:270–7. doi: 10.1111/j.1471-6402.2005.00221.x

[85] Woodlock D. The abuse of technology in domestic violence and stalking. Vio Against Women. (2017) 23:584–602. doi: 10.1177/1077801216646277, PMID: 27178564

[86] Kelly L Westmarland N (2015). Domestic violence perpetrator programmes: steps towards change. Project Mirabal final report. London and Durham: London Metropolitan University and Durham university. Available at: http://repository.londonmet.ac.uk/1458/ (Accessed June 28, 2023)

[87] Miragall M Baños RM Cebolla A Botella C. Working alliance inventory applied to virtual and augmented reality (WAI-VAR): psychometrics and therapeutic outcomes. Front Psychol. (2015) 6:1531. doi: 10.3389/fpsyg.2015.01531, PMID: 26500589 PMC4597032

[88] Gaston L. Reliability and criterion-related validity of the California psychotherapy Alliance scales—patient version. Psychol Assess. (1991) 3:68–74. doi: 10.1037/1040-3590.3.1.68

[89] Ritchie J Lewis J Nicholls CM Ormston R eds. Qualitative Research Practice: A Guide for Social Science Students and Researchers. London: Sage (2013).

[90] Neal JW Neal Z Van Dyke E Kornbluh M. Expediting the analysis of qualitative data in evaluation: a procedure for the rapid identification of themes from audio recordings (RITA). Am J Eval. (2014) 36:118–32. doi: 10.1177/1098214014536601

[91] Tong A Sainsbury P Craig J. Consolidated criteria for reporting qualitative research (COREQ): a 32-item checklist for interviews and focus groups. Int J Qual Health Care. (2007) 19:349–57. doi: 10.1093/intqhc/mzm042, PMID: 17872937

[92] Gilchrist G Johnson A Henderson J Dheensa S Radcliffe P Stephens-Lewis D. Understanding and reducing intimate partner violence perpetrated by men in substance use treatment: the ADVANCE research programme including a feasibility RCT and a non-randomised feasibility study. Program Grants Appl Res. (n.d.)

[93] Rempel E Donelle L Hall J Rodger S. Intimate partner violence: a review of online interventions. Inform Health Soc Care. (2019) 44:204–19. doi: 10.1080/17538157.2018.1433675, PMID: 29537928

[94] Easton CJ Crane C Mandel D. A randomized controlled trial assessing the efficacy of cognitive behavioural therapy for substance dependent domestic violence offenders: an integrated substance abuse-domestic violence treatment approach (SADV). J Marriage Fam Ther. (2018) 44:483–98. doi: 10.1111/jmft.12260, PMID: 29108096

[95] Kraanen FL Vedel E Scholing A Emmelkamp PMG. The comparative effectiveness of integrated treatment for substance abuse and partner violence (I-StoP) and substance abuse treatment alone: a randomized controlled trial. BioMed Central. (2013) 13:189–9. doi: 10.1186/1471-244X-13-189, PMID: 24059784 PMC3716952

[96] Hesser H Axelsson S Bäcke V Engstrand J Gustafsson T Holmgren E. Preventing intimate partner violence via the internet: a randomized controlled trial of emotion-regulation and conflict-management training for individuals with aggression problems. Clin Psychol Psychother. (2017) 24:1163–77. doi: 10.1002/cpp.2082, PMID: 28261923

[97] Turner W Morgan K Hester M Feder G Cramer H. Methodological challenges in group-based randomised controlled trials for intimate partner violence perpetrators: a meta-summary. Psychosoc Interv. (2023) 32:123–36. doi: 10.5093/pi2023a9, PMID: 37383642 PMC10294461

[98] Ferrer-Perez VA Bosch-Fiol E. Batterer intervention programs in Spain: an analysis of their effectiveness. Int J Offender Ther Comp Criminol. (2018) 62:885–97. doi: 10.1177/0306624X16672455, PMID: 27707933

[99] Clough A Wagman J Rollins C Barnes J Connor-Smith J Holditch-Niolon P. The SHARE project: maximizing participant retention in a longitudinal study with victims of intimate partner violence. Field Method. (2011) 23:86–101. doi: 10.1177/1525822X10384446

[100] Daly JE Pelowski S. Predictors of dropout among men who batter: a review of studies with implications for research and practice. Viol Victim. (2000) 15:137–60. doi: 10.1891/0886-6708.15.2.137, PMID: 11108498

[101] Lila M Gracia E Catalá-Miñana A. More likely to dropout, but what if they don’t? Partner violence offenders with alcohol abuse problems completing batterer intervention programs. J Interpers Viol. (2020) 35:1958–81. doi: 10.1177/0886260517699952 29294698

[102] Cunha O Silva A Cruz AR de Castro RA Braga T Gonçalves TA. Dropout among perpetrators of intimate partner violence attending an intervention program. Psychol Crime Law. (2023) 29:634–52. doi: 10.1080/1068316X.2022.2030337

[103] Olver ME Stockdale KC Wormith JS. A meta-analysis of predictors of offender treatment attrition and its relationship to recidivism. J Consult Clin Psychol. (2011) 79:6–21. doi: 10.1037/a0022200, PMID: 21261430

[104] Richards TN Jennings WG Murphy C. Risk and protective factors for batterer intervention treatment program attrition: how completers are distinct from dropouts and no-shows. J Interpers Violence. (2021) 36:7351–70. doi: 10.1177/0886260519834096, PMID: 30852952

[105] Cabral RR Smith TB. Racial/ethnic matching of clients and therapists in mental health services: a meta-analytic review of preferences, perceptions, and outcomes. J Couns Psychol. (2011) 58:537–54. doi: 10.1037/a0025266, PMID: 21875181

[106] Canidate S Hart M. The use of avatar counseling for HIV/AIDS health education: the examination of self-identity in avatar preferences. J Med Internet Res. (2017) 19:e365. doi: 10.2196/jmir.6740, PMID: 29196281 PMC5732328

[107] Gannon B Davis R Kuhns LM Rodriguez RG Garofalo R Schnall R. A Mobile sexual health app on empowerment, education, and prevention for young adult men (MyPEEPS Mobile): acceptability and usability evaluation. JMIR Format Res. (2020) 4:e17901. doi: 10.2196/17901, PMID: 32254043 PMC7175191

[108] Lee YH Xiao M Wells RH. The effects of avatars’ age on older adults’ self-disclosure and trust. Cyberpsychol Behav Soc Netw. (2018) 21:173–8. doi: 10.1089/cyber.2017.0451, PMID: 29638156

[109] Burbach P Pote H. Digital approaches—a paradigm shift? J Fam Ther. (2021) 43:169–84. doi: 10.1111/1467-6427.12336

[110] Torous J Jän Myrick K Rauseo-Ricupero N Firth J. Digital mental health and COVID-19: using technology today to accelerate the curve on access and quality tomorrow. JMIR Ment Health. (2020) 7:e18848. doi: 10.2196/18848, PMID: 32213476 PMC7101061

[111] Mark TL Treiman K Padwa H Henretty K Tzeng J Gilbert M. Addiction treatment and telehealth: review of efficacy and provider insights during the COVID-19 pandemic. Psychiatr Serv. (2022) 73:484–91. doi: 10.1176/appi.ps.202100088, PMID: 34644125

